# Omental whirl associated with bilateral inguinal hernia: a case report

**DOI:** 10.1186/1752-1947-8-239

**Published:** 2014-07-01

**Authors:** Elsa Silva, Ana Franky Carvalho, Diogo Rocha, António Mesquita Rodrigues, Ricardo Pereira, Ana João Rodrigues, Pedro Leão

**Affiliations:** 1 General Surgery, Hospital of Braga, 4701-965, Braga, Apartado 2242, Portugal; 2 Life and Health Sciences Research Institute (ICVS), School of Health Sciences, University of Minho, Braga, Portugal; 3 ICVS/3B’s - PT Government Associate Laboratory, Braga/Guimarães, Portugal; 4 Radiology Department, Hospital of Braga, 4701-965 Braga, Portugal

**Keywords:** Abdominal pain, Omentum, Torsion, Whirl sign

## Abstract

**Introduction:**

Torsion of the omentum is a rare cause of abdominal pain. It is clinically similar to common causes of acute surgical abdomen and is often diagnosed during surgery. Inguinal hernia is a common condition but not frequently related with torsion of the omentum.

**Case presentation:**

A 40-year-old Caucasian man came to our emergency department with abdominal pain of the left quadrant and abdominal distension for 2 days. His medical history included an untreated left inguinal hernia in the last year. Computed tomography revealed densification of mesocolon with left omentum “whirl” component and other signs of omental torsion. During an exploratory laparoscopy, a wide twist of his omentum with necrotic alterations that extended to the bilateral inguinal hernial content was observed. Omentectomy and surgical repair of bilateral inguinal hernia were performed.

**Conclusions:**

Torsion of the omentum is a rare entity and usually presents a diagnostic challenge. The use of abdominal computed tomography can help diagnosing torsion of the omentum preoperatively and, thus, prevents a surgical approach. Nonetheless, some cases of torsion of the omentum require surgical repair. Accordingly, a laparoscopic approach is minimally invasive and efficient in performing omentectomy.

## Introduction

Torsion of the omentum is a clinical condition in which the organ rotates on its long axis compromising its vascularity. Segmental torsion of the greater omentum is more common; it was first described by Bush in 1896 [1]. By 2001, slightly fewer than 300 cases had been reported, almost all misdiagnosed as acute appendicitis and discovered during an exploratory laparotomy [2,3]. The condition is more common in males, with a ratio of 2:1 in the third decade of life and 5:1 in the fourth decade of life [1,4], but it can occur at any age and it can be primary or secondary [5]. Primary torsion (idiopathic) is less common [1], whereas secondary torsion is associated with pre-existing conditions including longer than normal omentum, internal hernias, inflammatory pathologies of other organs such as acute cholecystitis, pancreatitis, and adnexitis, tumors, and postsurgical adhesions [1]. There are also predisposing factors such as sex, obesity, sudden strong increase in intra-abdominal pressure (brought on by coughing or violent exercise), traumas, autonomics (large pedicle, larger or more twisted than normal epiploic blood vessels), hyperperistalsis, surgical adherences, or some acute process in an intracavitary organ causing displacement of the omentum [5,6].

Torsion of the omentum presents nonspecific clinical symptoms, namely moderate abdominal pain that may be similar to acute appendicitis. Thus, it is difficult to obtain a preoperative clinical diagnosis. According to the literature, ultrasound and computed tomography (CT) are useful tools for establishing a preoperative diagnosis [7,8].

The purpose of this publication is to present a case report of a patient with a rare condition of unusual clinical presentation. We report a case of torsion of the greater omentum diagnosed preoperatively associated with bilateral inguinal hernias. Moreover, we also show that minimally invasive surgery can help to confirm the diagnosis, while providing treatment and a better postoperative recovery.

## Case presentation

A 40-year-old Caucasian man was admitted to our emergency department with moderate pain of his left abdomen for 2 days. He also complained of abdominal distension but presented no other signs and symptoms like fever, nausea, vomiting, diarrhea or intestinal obstruction. His medical history included an untreated left inguinal hernia in the last year. A physical examination revealed pain in his left abdomen associated with a palpable mass on his left flank, without rebound tenderness or guarding.

**Figure 1:**
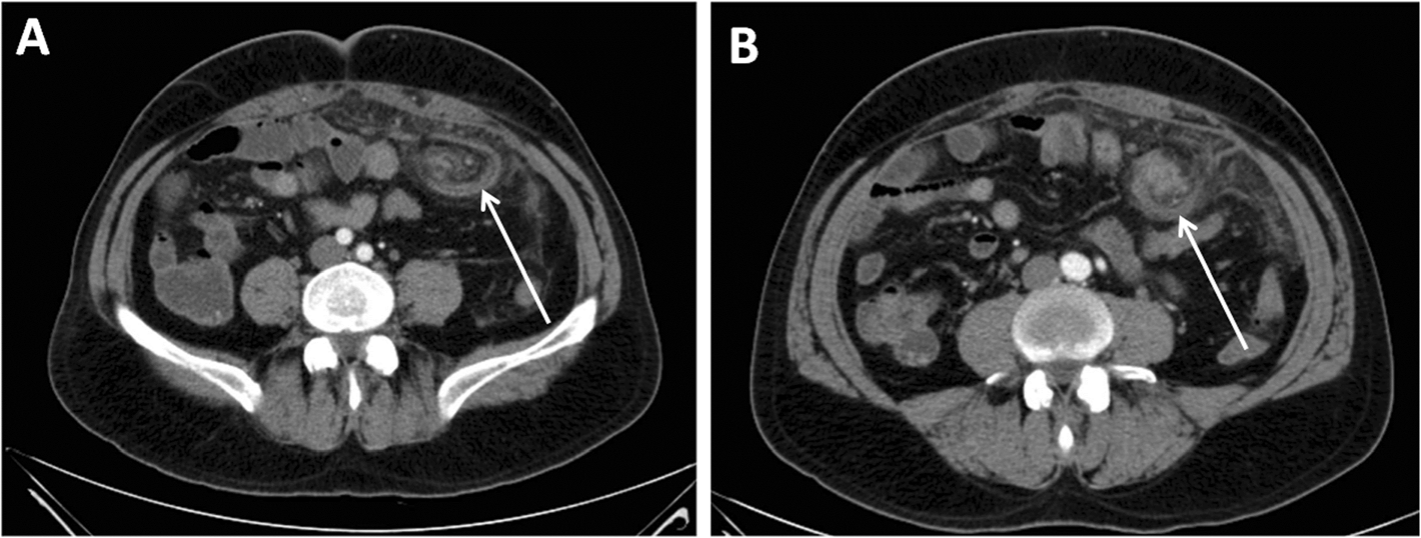
**Computed tomography scans showing the whirl (arrow) of the omentum. A)** caudal view. **B)** cranial view.

Two bilateral reducible inguinal hernias were found. Laboratory results revealed a C-reactive protein of 124mg/L. Given this unspecific clinical presentation, an abdominal and pelvic CT scan were performed, revealing an intra-abdominal abnormality, suggestive of pathologic infiltration of his omentum and the presence of the whirl sign suggesting torsion of his greater omentum (Figure 1A and 1B). The distal end of his omentum seemed to be alongside the spermatic cord into a bilateral inguinal hernia sac. Since there was no evidence of acute abdomen or incarcerated hernia and he was stable, we decided on conservative treatment because it has been reported that spontaneous derotation of the omentum can occur [9]. After he had spent 2 days as an in-patient of our hospital, his pain persisted and his left inguinal hernia became incarcerated. A preoperative diagnosis of secondary torsion of his greater omentum associated with an inguinal bilateral hernia (particularly left inguinal hernia) was established and he submitted to surgery.

**Figure 2:**
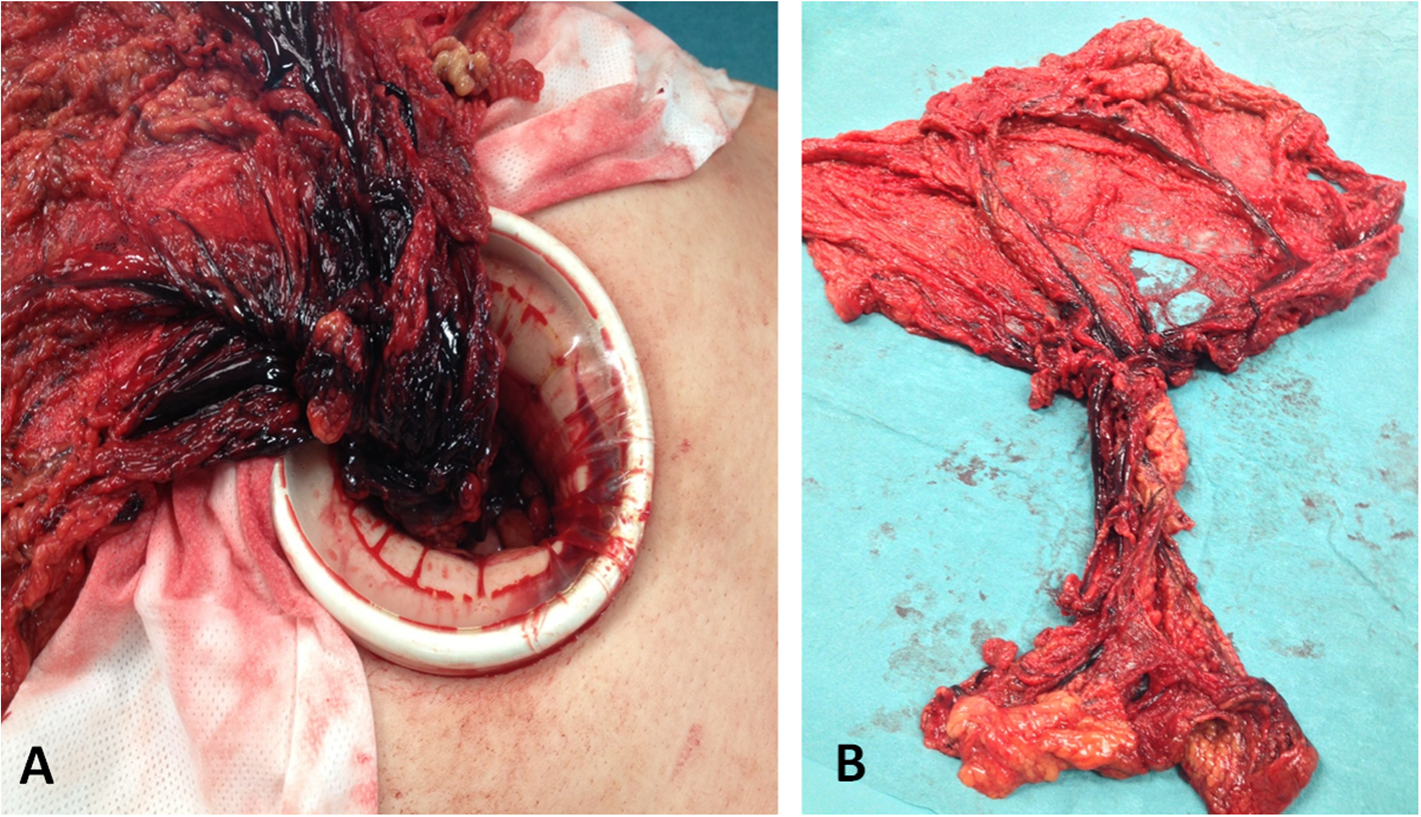
**Intraoperatory view of the whirl of the omentum. A)** Exteriorization of the omentum after minilaparotomy showing the precise point of torsion. **B)** Complete exeresis of the twisted omentum.

Laparoscopy was performed to evaluate the extent of the damage to his omentum. A wide twist of omentum with large necrotic vascular changes that extended to the inguinal hernia content was found (Figure 2A). After reducing the hernia content, omentectomy was performed. His abdominal cavity was reached using a 10mm trocar under his umbilicus. Two accessory trocars were placed in his right flank (5mm). A median minilaparotomy (6cm) through the umbilicus port was created for exteriorization and resection of his large necrotic omentum (Figure 2B). Surgical repair of bilateral inguinal hernia was performed by Lichtenstein technique, since the success of laparoscopic inguinal hernia repair techniques (such as transabdominal preperitoneal) in incarcerated hernias is still controversial [10,11]. A histopathological examination revealed large areas of interstitial hemorrhage, vascular congestion and thrombosis compatible with omental torsion.

His postoperative period occurred without complications and he was discharged on the third day after surgery. One month later he yielded no complaint.

## Discussion

Omental torsion is a benign rare cause of acute abdomen, easily misdiagnosed as acute appendicitis, acute cholecystitis or diverticulitis. It is defined as axial twisting along the long axis of the omentum to such an extent that its vascularity is compromised. It most frequently affects the right side because the right side of the omentum is longer than the left side, more mobile and less richly vascularized with poor collateralization [12,13]. However, in this case, we found a complete torsion of the greater omentum with necrosis secondary to incarceration in a bilateral inguinal hernia, more evident on the left side. The predisposing factors identified in this case were sex (more frequent in males), a large pedicle and history of untreated inguinal hernia. Given the few clinical signs, the preoperative diagnosis was largely based on radiological findings. CT findings of greater omental torsion include a well circumscribed, oval, or cake-like fatty mass with heterogeneous attenuation, containing strands of soft tissue attenuation [12] and particularly the presence of concentric linear strands (the “whirl sign”). Of notice, the whirl sign may not be as apparent if the axis of rotation is not perpendicular to the transverse scanning plane [14]. Although a CT scan is helpful in diagnosing torsion of the omentum and may prevent an unnecessary surgery, the extension of omental torsion may not be clearly visualized through this technique, rendering close vigilance of clinical resolution/deterioration. Segmental omental torsion is usually a benign and self-limiting disease [15], capable of evolving to resolution within 2 weeks with conservative measures. This fact was taken into consideration since the patient experienced no clinical signs of deterioration in the emergency service. The treatment of complete torsion of the greater omentum with secondary necrosis is usually surgical [8]. In this case, a laparoscopic approach should be considered because it is less invasive and associated with lower morbidity. Moreover, when preoperative diagnosis is not clear by imaging techniques, laparoscopy is useful for both diagnosis and treatment [13,16]. The criticism to this approach is the surgical treatment that was used for the treatment of the hernias, since laparoscopic repair of incarcerated, non-reducible groin hernias has to be done urgently and can be performed with an endoscopic technique, as advised by the European Association for Endoscopic Surgery [17]. However, a surgeon should not endanger the patient's life. In this perspective, there is controversy regarding the laparoscopic approach to large incarcerated inguinal hernias.

The combination of imaging techniques and minimally invasive surgery were helpful in making a correct diagnosis and avoiding a more invasive surgical approach such as laparotomy.

## Conclusions

Conservative treatment of primary torsion has been described in cases of partial omental torsion. However, the treatment of complete torsion of the greater omentum with secondary necrosis is surgical. The presence of precipitating factors for torsion of the omentum (secondary torsion) should render close vigilance of the patient as the situation can worsen. It is still not clear whether treatment of the precipitating factor should be the initial approach. Nonetheless, the use of a laparoscopic approach seems to be successful in such a benign situation.

## Consent

Written informed consent was obtained from the patient for publication of this case report and any accompanying images. A copy of the written consent is available for review by the Editor-in-Chief of this journal.

## Competing interests

The authors declare that they have no competing interests.

## Authors’ contributions

ES and PL evaluated the patient. AFC, AJR, AMR and PL were involved in drafting the manuscript and revising it critically for important intellectual content. DR was involved in CT scanning and imaging evaluation. PL, RP and ES performed the laparoscopic omentectomy and hernia repair. PL and AMR were involved in revising it critically and have given final approval of the version to be published. All authors read and approved the final manuscript
